# Layer-by-Layer Combination of MWCNTs and Poly(ferulic acid) as Electrochemical Platform for Hesperidin Quantification

**DOI:** 10.3390/bios13050500

**Published:** 2023-04-25

**Authors:** Elvira Yakupova, Aisylu Mukharlyamova, Igor Fitsev, Guzel Ziyatdinova

**Affiliations:** 1 Analytical Chemistry Department, Kazan Federal University, Kremleyevskaya, 18, Kazan 420008, Russia; 2 Federal State Budgetary Scientific Institution «Federal Center for Toxicological, Radiation, and Biological Safety», Nauchny Gorodok-2, Kazan 420075, Russia

**Keywords:** voltammetric sensors, chemically modified electrodes, carbon nanomaterials, electropolymerization, phenolic acids, flavanones, hesperidin

## Abstract

The electrochemical polymerization of suitable monomers is a powerful way to create voltammetric sensors with improved responses to a target analyte. Nonconductive polymers based on phenolic acids were successfully combined with carbon nanomaterials to obtain sufficient conductivity and high surface area of the electrode. Glassy carbon electrodes (GCE) modified with multi-walled carbon nanotubes (MWCNTs) and electropolymerized ferulic acid (FA) were developed for the sensitive quantification of hesperidin. The optimized conditions of FA electropolymerization in basic medium (15 cycles from −0.2 to 1.0 V at 100 mV s^−1^ in 250 µmol L^−1^ monomer solution in 0.1 mol L^−1^ NaOH) were found using the voltammetric response of hesperidin. The polymer-modified electrode exhibited a high electroactive surface area (1.14 ± 0.05 cm^2^ vs. 0.75 ± 0.03 and 0.089 ± 0.003 cm^2^ for MWCNTs/GCE and bare GCE, respectively) and decreased in the charge transfer resistance (21.4 ± 0.9 kΩ vs. 72 ± 3 kΩ for bare GCE). Under optimized conditions, hesperidin linear dynamic ranges of 0.025–1.0 and 1.0–10 µmol L^−1^ with a detection limit of 7.0 nmol L^−1^ were achieved, which were the best ones among those reported to date. The developed electrode was tested on orange juice and compared with chromatography.

## 1. Introduction

The electrochemical polymerization of suitable monomers is a powerful way to create voltammetric sensors with improved responses to a target analyte [1,2,3,4]. Major attention has been focused on conductive polymers such as polyaniline [1,5], polypyrrole [3], polythi-ophene [1,6], aminobenzoic acids [7,8], and their derivatives. On the other hand, nonconductive polymers, which are based on electropolymerized phenols [9], aminophenols [10,11], and natural phenolics [12], have been successfully used in biosenors and chemical sensors.

Among a wide range of natural phenolics, hydroxycinnamic acids are of interest as monomers. Their electropolymerization can follow various schemes depending on the monomer structure and the conditions of the electrochemical process [12]. Therefore, a great variety of polymers with unique properties can be obtained and used for the creation of electrochemical sensors and biosensors.

Contrary to conductive polymers, poly(hydroxycinnamic acids) show low capacitive currents that significantly improve the shape of target analytes’ voltammograms and the accuracy of their determination. Furthermore, electropolymerization takes less time, as it is self-limited. Due to the small thickness of such coatings, the target analytes and their oxidation/reduction products can easily diffuse to/from the modified electrode surface, thereby decreasing their response times and improving the selectivity of analyte determination. Nevertheless, there is a lack of investigations of hydroxycinnamic acid electropolymerization compared to other natural phenolics. The potentiodynamic electropolymerization of ferulic acid (FA) and the immobilization of glucose oxidase onto a carbon paste electrode have been carried out in a single step to create a glucose biosensor [13]. Several electrochemical sensors based on electropolymerized caffeic acid have been described for the simultaneous voltammetric determination of ascorbic acid, epinephrine, and uric acid [14]; ascorbic acid and dopamine [15]; copper and lead [16]; as well as the individual quantification of L-3,4-dihydroxyphenylalanine (L-DOPA) [17], metanephrine [18], reduced nicotinamide adenine dinucleotide (NADH) [19], acetaminophen [20], and chloramphenicol [21]. Poly(*p*-coumaric acid)-modified graphite electrodes allow for the simultaneous determination of cadmium and lead [22]. Electrodeposition of the polymer is performed on the glassy carbon electrode (GCE) using a potentiodynamic mode.

Further development in the field has been focused on the improvement of polymer-modified electrode characteristics. The insulating nature of polymers significantly affects electrode conductivity. Therefore, layer-by-layer combinations of such coverages with the carbon nanomaterials as a platform for polymer electrodeposition has been applied to provide sufficient conductivity and high surface area to the electrode [12]. Furthermore, such an approach makes it possible to obtain more uniform coverage and a higher load on the electrode surface [23].

A few data (Table 1) reported for the sensors with poly(hydroxycinnamic acids) layers combined with carbon nanomaterials confirm the effectiveness of this approach for electrode surface modification.

FA electropolymerization is less studied compared to other hydroxycinnamic acids. Furthermore, the process has been performed in acidic mediums, and electroactive coverage has been obtained due to the formation of quasi-reversible redox pairs of oxidized/reduced quinones [28]. Similar to caffeic acid [26], electropolymerization involves the double bond and electrode acting on the principles of electrocatalysis [29]. As is known [12,27], the electropolymerization of phenolic compounds is better carries out in a basic medium due to easier detachment of the electron from the anion existing under these conditions.

Another aspect of further developments on this topic is the enlargement of the analytes studied. Contrary to the electrodes based on poly(hydroxybenzoic acids) [30,31,32,33], sensors with electropolymerized hydroxycinnamic acids have never been used for the determination of natural phenolic compounds.

Hesperidin (Figure S1) is a natural flavonoid of citrus fruits, which is widely used in medicine for blood vessel disorders treatment such as hemorrhoids, varicose veins, and impaired circulation [34]. Hesperidin is an electroactive compound because of the presence of phenolic fragments in its structure. However, few studies on the electrochemical determination of hesperidin have been presented to date, and the analytical characteristics are not impressive.

The current work deals with the development of novel voltammetric sensors based on layer-by-layer combinations of multi-walled carbon nanotubes (MWCNTs) and poly(FA) that was obtained electrochemically in the basic medium. The conditions of voltammetric sensor fabrication were found using hesperidin as a target analyte. The electrode was characterized by scanning electron microscopy and electrochemical methods. The electrooxidation parameters of hesperidin were calculated. A highly sensitive and selective voltammetric sensor for hesperidin was developed and tested on orange juice.

## 2. Materials and Methods

### 2.1. Reagents

Hesperidin (94% purity) was purchased from Sigma (Steinheim, Germany). Its 0.40 mmol L^−1^ stock solution was prepared in methanol (c.p. grade). FA (99%) from Aldrich (Steinheim, Germany) was used as a monomer for the electrode surface modification. Its 10 mmol L^−1^ stock solution was prepared in methanol. MWCNTs (o.d. 40–60 nm, i.d. 5–10 nm and *l* = 0.5–500 μm) from Aldrich (Steinheim, Germany) were used as a platform for further electrodeposition of polyFA. Ascorbic (99%), gallic (99%), caffeic (98%) acids, naringin (95%), and quercetin (95%) from Sigma (Steinheim, Germany), chlorogenic acid (95%) from Aldrich (Steinheim, Germany), and rutin trihydrate (97%) from Alfa Aesar (Heysham, UK) were used in the interference study. Their 10 mmol L^−1^ stock solutions were prepared in methanol. An exact dilution was used to obtain less concentrated solutions.

HPLC-grade acetonitrile was obtained from Panreac (Barcelona, Spain). Water for chromatographic measurements was deionized in a Milli-Q purification system (Millipore Corporation, Bedford, MA, USA).

Other reagents were of c.p. grade and were used without additional treatment. Distilled water was used for the preparation of supporting electrolytes in voltammetry.

### 2.2. Equipment

Potentiostats/galvanostats Autolab PGSTAT 12 and Autolab PGSTAT 302N with the FRA 32M module (Eco Chemie B.V., Utrecht, The Netherlands) supplied with the NOVA 1.10.1.9 software (Eco Chemie B.V., Utrecht, The Netherlands) and glass electrochemical cells were used in the electrochemical measurements. The three-electrode system consisted of the working electrode (GCE of 3 mm diameter (BASi^®^ Inc., West Lafayette, IN, USA) or a modified electrode (MWCNTs/GCE or polyFA/MWCNTs/GCE)), an Ag/AgCl reference electrode, and a platinum wire as the auxiliary electrode.

An “Expert-001” pH meter (Econix-Expert Ltd., Moscow, Russia) with a glassy electrode was used for supporting electrolyte pH measurements.

An ultrasonic bath (WiseClean WUC-A03H (DAIHAN Scientific Co., Ltd., Wonju-si, Republic of Korea) was used for the MWCNTs’ suspension and sample preparation.

An Agilent 1100 Series HPLC equipped with diode-array detector and autosampler (Agilent Technologies, Waldbronn, Germany) was used. A Luna 100 18 column (25 cm × 4.6 mm, 2.5 µm) from Phenomenex (Torrance, CA, USA) was used for the separation.

A Merlin^TM^ high-resolution field emission scanning electron microscope (Carl Zeiss, Oberkochen, Germany) was operated at a 5 kV accelerating voltage and a 300 pA emission current.

### 2.3. Experimental Conditions

#### 2.3.1. Electrochemical Measurements

Bare GCEs were mechanically polished on alumina slurry with particle size of 0.05 µm and thoroughly washed with acetone and distilled water. The platinum electrode was cleaned in nitric acid (1:1) for 3 min and then thoroughly washed with distilled water.

For GCE modification, 0.5 mg mL^−1^ homogeneous suspension of MWCNTs was prepared in 1% sodium dodecyl sulfate (Panreac, Barcelona, Spain) by sonication for 30 min in an ultrasonic bath. Then, 5 µL of a MWCNT suspension was drop casted on the electrode surface and evaporated to dryness for 15 min.

Potentiodynamic electropolymerization of FA on the MWCNTs/GCE was performed under various conditions using voltammetric characteristics of hesperidin as indicating parameters. Five cycles of supporting electrolyte were recorded prior to FA addition in the electrochemical cell and its electropolymerization.

The electrode surface was renewed after each measurement by mechanical removal of both MWCNT and poly(ferulic acid) layers.

Voltammetric measurements of hesperidin were performed in 0.1 mol L^−1^ phosphate buffer of various pH. Prior to measurement with analyte, five curves of the supporting electrolyte were recorded. Then, an aliquot portion of hesperidin solution was added to the electrochemical cell (the total volume of solution in the cell was 4.0 mL) and voltammograms were recorded in the range of 0.3–1.3 V for cyclic voltammetry and 0.2–1.2 V for differential pulse mode. Potential scan rate and pulse parameters were varied. Baseline-correction in NOVA 1.10.1.9 software (Eco Chemie B.V., Utrecht, The Netherlands) was used for differential pulse voltammetry.

Chronoamperometric evaluation of bare GCE electroactive surface area was performed in 0.1 mol L^−1^ KCl using 1.0 mM ferrocyanide ions as a standard. Constant potential of 0.45 V and electrolysis time of 75 s were used.

A 1.0 mmol L^−1^ mixture of ferro-/ferricyanide ions in 0.1 mol L^−1^ KCl was used as a redox probe in the electrochemical impedance spectroscopy. The impedance spectra were recorded in the frequency range of 10 kHz–0.04 Hz with an applied sine potential amplitude of 5 mV at a polarization potential of 0.21 V, which was calculated as a half-sum of the redox peaks of ferro-/ferricyanide ions mixture in cyclic voltammetry.

#### 2.3.2. Orange Juice Analysis by Voltammetry

Fresh and commercially available orange juice was used in the study. Fresh juice was prepared by cutting the fruit in half and carefully hand-squeezing. Both types of juices were diluted with methanol in a 1:1 ratio (6.0 mL of juice was used) and sonicated for 15 min in an ultrasonic bath [35]. Then, the solution obtained was filtered through nylon membrane filters of 0.45 µm pore size (Agilent Technologies, Inc., Santa Clara, CA, USA).

A total of 10 µL of the treated juice were inserted into the electrochemical cell containing 0.1 mol L^−1^ phosphate buffer with pH 5.5, and differential pulse voltammograms were recorded from 0.2 to 0.9 V at the pulse amplitude of 0.10 V, pulse time of 0.025 s, and potential scan rate of 20 mV s^−1^. Oxidation currents of hesperidin were calculated using the baseline correction in NOVA 1.10.1.9 software.

#### 2.3.3. Chromatographic Determination of Hesperidin in Orange Juices

The chromatographic quantification of hesperidin was performed as described in [35]. Briefly, the treated juice (Section 2.3.2.) was studied using a linear gradient elution with water and acetonitrile. The following gradient elution program was used (Table 2) at the flow rate of 1 mL min^−1^.

The column temperature was kept at 25 °C. The sample injection volume was 20 µL. Hesperidin retention time was 17.04 min. Quantification was performed at 340 nm using a calibration graph.

### 2.4. Statistical Analysis

Electrochemical measurements were performed in five replications, and chromatographic measurements were performed in three replications. The average value of the parameters and the corresponding coverage interval at α = 0.05 were used for data presentation. Relative standard deviation (RSD) was used for random error characterization. *F*- and *t*-tests were applied for the validation of the method developed.

OriginPro 8.1 software (OriginLab, Northampton, MA, USA) was used for regression and statistical analysis.

## 3. Results and Discussion

### 3.1. Electrodeposition of PolyFA on the MWCNTs/GCE

**Figure 1:**
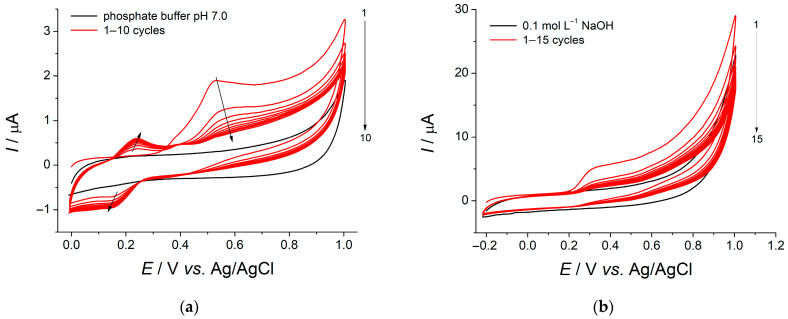
Cyclic voltammograms of ferulic acid (FA) on glassy carbon electrode modified with multi-walled carbon nanotubes (MWCNTs/GCE): (**a**) 100 µmol L^−1^ FA in phosphate buffer pH 7.0; (**b**) 250 µmol L^−1^ FA in 0.1 mol L^−1^ NaOH. υ = 100 mV s^−1^.

FA oxidation on the MWCNTs/GCE was studied in neutral and basic media (Figure 1). An oxidation peak at 0.52 V was observed on the first scan in the phosphate buffer with a pH of 7.0, which gradually shifted to a positive potential and decreased in currents on the following cycles (Figure 1a). The reversible quinone/hydroquinone redox pair at 0.16/0.23 V formed on the cathodic branch of the first cycle and following the anodic scan of the second cycle. Its redox currents increased with the increase in the number of cycles, thus confirming the formation of electroactive coverage similar to that obtained in the acidic medium [28].

**Scheme 1:**
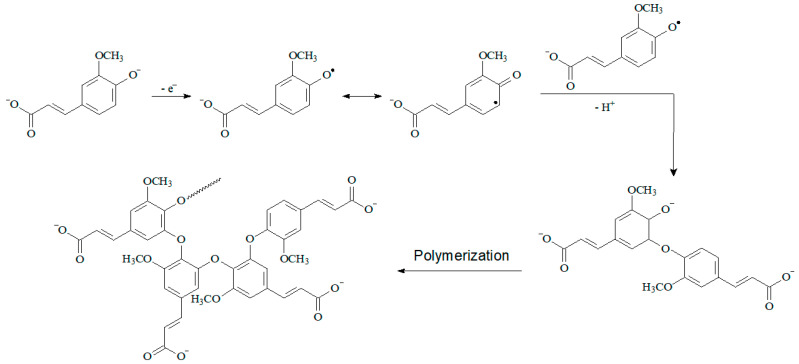
FA electropolymerization in a basic medium.

A totally different behavior of FA at the same electrode was observed in the basic medium. An irreversible oxidation peak at 0.31 V was observed (Figure 1b), which decreased as the number of cycles increased. Such behavior indicates that another type of electrochemical reaction occurred. The process involves one electron detachment from the phenolate ion existing in the basic medium (the pKa of the FA phenolic group was 8.92 [36]) with the formation of a phenoxyl radical (Scheme 1), which undergoes further reactions of dimerization and polymerization similar to *p*-coumaric acid [12]. These data confirmed the formation of the insulating coverage and agree well with the data reported for hydroxybenzoic [30,31,32,33] and other hydroxycinnamic acids [22,27]. The polymeric coverage obtained does not show electrochemical activity to allowing the use of its own response of the target compound for analytical purposes.

**Figure 2:**
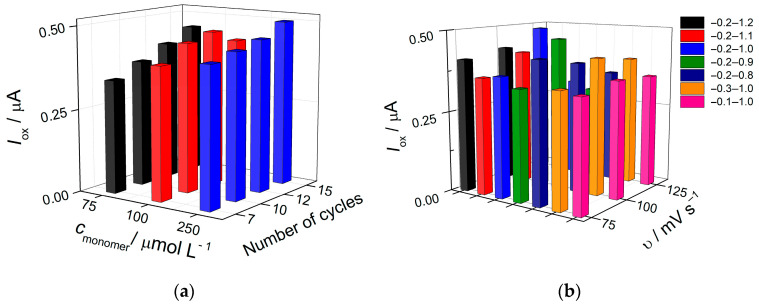
Effect of the FA electropolymerization conditions on the 10 µmol L^−1^ hesperidin oxidation currents on polyFA-modified electrode. (**a**) Effect of monomer concentration and number of scans. (**b**) Effect of electrochemical window and potential scan rate.

Target analyte responses on the polymer-modified electrode depend on the conditions of electropolymerization [12]. Therefore, the optimization of the electropolymerization conditions was performed using the voltammetric characteristics of hesperidin obtained in differential pulse mode in a phosphate buffer with a pH of 7.0. The effect of monomer concentration and number of cycles, as well as electrochemical window and potential scan rate, reflecting the electrolysis time effect, are shown (Figure 2). Hesperidin oxidation potential was almost the same, independent of the electropolymerization conditions mentioned above. The oxidation currents changed statistically significantly, and were used to optimize the FA electropolymerization conditions.

As one can see from Figure 1a, an increase in the monomer concentration and number of cycles provided an improvement of the hesperidin response. The highest oxidation currents were obtained on the polymeric coverage obtained from the 250 µmol L^−1^ monomer using 15 cycles. Further increases in the monomer concentration and number of cycles did not provide a significant increase of the hesperidin oxidation currents, due to the higher thickness of the polymeric coverage.

Variation in the electrolysis parameters showed that the higher oxidation currents of hesperidin were registered on the polymeric layers obtained at the potential scan rate of 100 mV s^−1^, which were independent of the electrochemical window. Among them, the best response of the hesperidin was observed for the electrochemical window from −0.2 to 1.0 V. These data confirm the effect of the electrolysis time on the properties of the polymeric coverage with respect to its thickness.

Thus, electrodes with the polyFA layer obtained by 15 cycles from −0.2 to 1.0 V with a scan rate of 100 mV s^−1^ in 250 µmol L^−1^ monomer solution were used in further studies.

### 3.2. Characterization of Bare and Modified GCE Using Scanning Electron Microscopy, Voltammetry, and Electrochemical Impedance Spectroscopy

**Figure 3:**
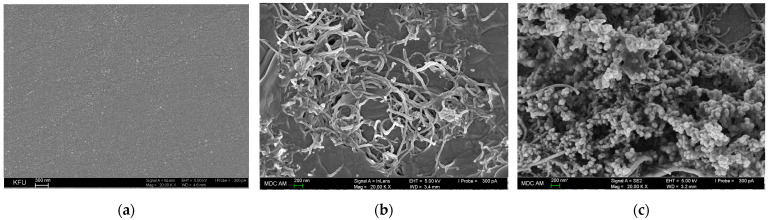
Electrode surface morphology based on scanning electron microscopy: (**a**) bare GCE; (**b**) MWCNTs/GCE; (**c**) PolyFA/MWCNTs/GCE. Magnification 20 K×.

Scanning electron microscopy was applied for the electrodes’ morphology characterization (Figure 3). The data obtained clearly indicate the even distribution of MWCNTs (Figure 3b) and polymer (Figure 3c) on the electrode surface. The PolyFA consisted of spherical particles of 90–120 nm diameter that covered the MWCNTs net. These data are in agreement with the morphology of poly(ellagic acid)/MWCNTs-modified electrodes [33].

**Figure 4:**
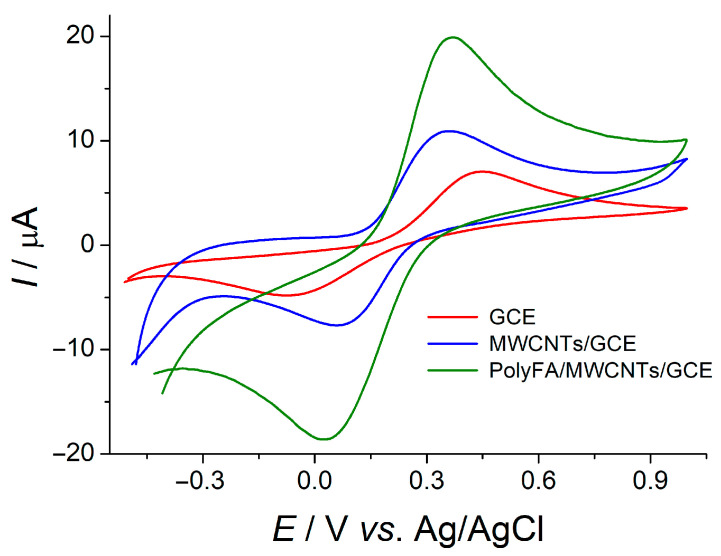
Cyclic voltammograms of 1.0 mM ferrocyanide ions in 0.1 mol L^−1^ KCl. υ = 100 mV s^−1^.

The cyclic voltammetry of ferrocyanide ions in 0.1 mol L^−1^ KCl was used for the evaluation of the electroactive surface area (Figure 4). Comparison of the cyclic voltammograms for bare GCE, MWCNTs/GCE, and polyFA/MWCNTs/GCE clearly indicated an improvement in the electrochemical system reversibility, as well as a statistically significant increase of the redox currents. This trend is in line with the data reported for poly(*p*-coumaric acid)- [27], poly(gallic acid)- [30], and poly(ellagic acid)- [33] based electrodes. The calculation of the electroactive surface area using the Randles–Ševčík equation showed a significant increase with the addition of each modifying layer (0.089 ± 0.003 cm^2^ for bare GCE, 0.75 ± 0.03 cm^2^ for MWCNTs/GCE, and 1.14 ± 0.05 cm^2^ for polyFA/MWCNTs/GCE) that agreed with SEM results.

**Figure 5:**
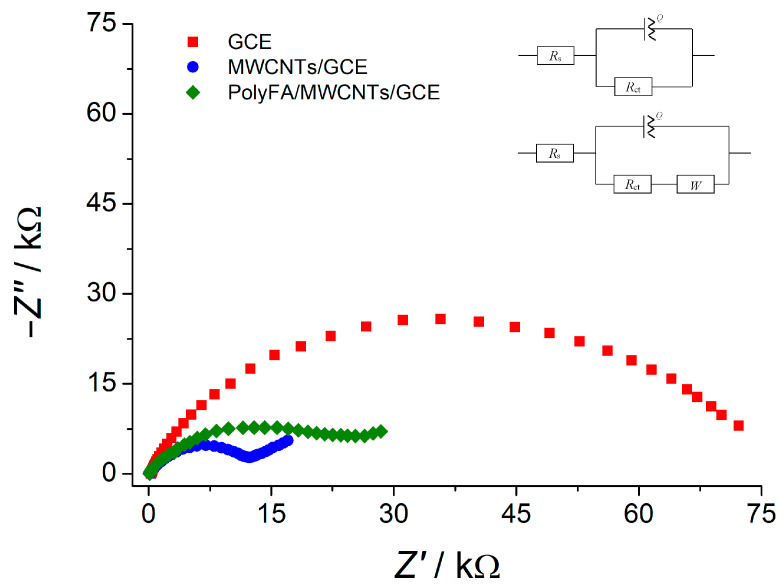
Nyquist plot for GCE, MWCNTs/GCE, and polyFA/MWCNTs/GCE recorded in the presence of 1.0 mmol L^−1^ mixture of ferro-/ferricyanide ions in 0.1 mol L^−1^ KCl at 0.21 V in the frequency range of 10 kHz–0.04 Hz with an applied sine potential amplitude of 5 mV. Insert is the Randles’ equivalent circuits used for impedance spectra fitting.

Electrochemical impedance spectroscopy in the presence of a redox probe (1.0 mmol L^−1^ mixture of ferro-/ferricyanide ions in 0.1 mol L^−1^ KCl) at 0.21 V was applied for the estimation of the electrodes’ electron transfer properties. The corresponding data are presented as Nyquist plots in Figure 5.

The semicircle diameter observed in the Nyquist plots at high frequencies was significantly different for the modified electrodes. This behavior means lower charge transfer resistance for the modified electrodes. The comparison of spectra for MWCNTs/GCE and polyFA/MWCNTs/GCE showed an increase in the charge transfer resistance for polymer-modified electrode that was caused by the insulating properties of the polyFA layer. A similar effect was obtained for other polymeric coverages based on hydroxybenzoic [30,32,33] and *p*-coumaric [27] acids. The quantitative parameters of impedance spectra were obtained by fitting with the Randles’ equivalent circuits consisting of the electrolyte resistance (*R*_s_), the charge transfer resistance (*R*_ct_), the constant phase element (*Q*), and the Warburg impedance (*W*) [37]. Taking into account the shape of the Nyquist plot for the GCE, no Warburg impedance was used in the equivalent circuit. Quantitative data of the impedance are presented in Table 3.

The PolyFA-modified electrode showed a 3.4-fold decrease in the charge transfer resistance vs. the bare GCE, which indicated an increase in the electron transfer rate. The non-conducting properties of polymeric coverage lead to a 1.8-fold higher charge transfer resistance compared to the MWCNTs/GCE, which agrees with data for polymers based on other phenolic acids [27,30,32,33]. The heterogeneous rate constant (*k*_et_) for the redox probe on the electrodes was calculated from the data obtained using Equation (1) [38]

*k*_et_ = *RT*/*F*^2^*n*^2^*R_ct_Ac*, (1)

where *R* is the universal gas constant (8.314 J mol^−1^ K^−1^), *T* is the temperature (298 K), *F* is the Faraday constant (96485 C mol^−1^), *n* is the number of electrons, *R_ct_* is the charge transfer resistance (Ω), *A* is the electrode surface area (cm^2^), and *c* is the redox probe concentration in the impedance measurements (mol cm^−3^). The *k*_et_ values of 5.23 × 10^−5^, 3.11 × 10^−4^, and 1.75 × 10^−4^ cm s^−1^ were obtained for the bare GCE, MWCNTs/GCE, and polyFA/MWCNTs/GCE, respectively.

The constant phase element was approximately 1.6-fold higher than that for bare GCE and MWCNTs/GCE, which was probably caused by the porous structure of the electrode surface and indirectly confirmed by the *n* value. A significant decrease in the solution resistance for polyFA/MWCNTs/GCE is explained by the porosity of the electrode surface. The permeability of the electrode surface to the electrolyte solution increases with increasing porosity [37].

Thus, the polyFA-modified electrode showed favorable properties for application in electroanalysis.

### 3.3. Voltammetric Characteristics of Hesperidin on Bare and Modified GCE

**Figure 6:**
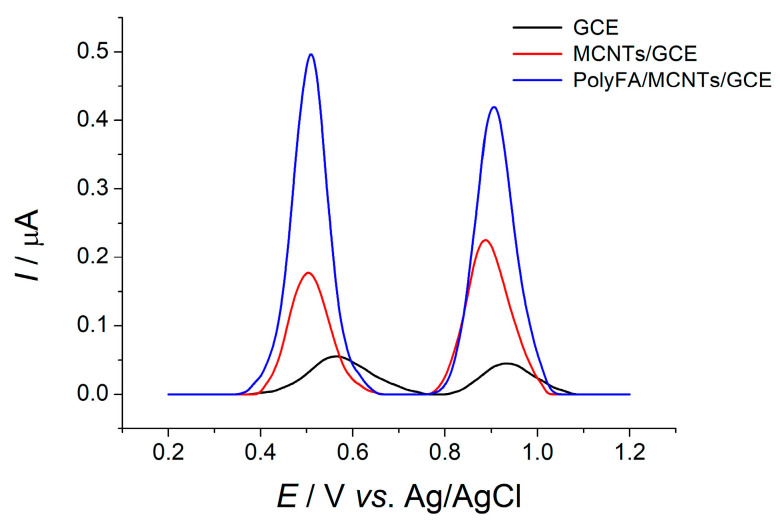
Differential pulse voltammograms with baseline correction for 10 µmol L^−1^ hesperidin on the bare GCE and modified electrodes in phosphate buffer pH 7.0. Δ*E*_pulse_ = 0.050 V, *t*_pulse_ = 0.050 s, υ = 20 mV s^−1^.

Hesperidin is electroactive on the bare GCE in a phosphate buffer with a pH of 7.0. Well-resolved oxidation peaks at 0.57 and 0.93 V were observed on the differential pulse voltammograms (Figure 6). The oxidation currents of 0.055 ± 0.002 and 0.046 ± 0.002 for the first and second steps, respectively, were registered for a 10 µmol L^−1^ concentration, thereby indicating an insufficient sensitivity of the electrode response. Furthermore, the first peak had a stretched shape that affected the determination of hesperidin in real samples.

Similar to other flavanones [33,39,40], the electrode surface modification with MWCNTs provided a shift in the hesperidin oxidation potential to lower values on 64 and 47 mV for the first and second steps, respectively. Oxidation currents were significantly increased (0.18 ± 0.01 and 0.22 ± 0.01 µA for the first and second peak, respectively) due to the higher electroactive surface area of the modified electrode. The first oxidation peak was less than the second one, thus making it less sensitive for quantification purposes.

The PolyFA-based electrode also showed two well-pronounced oxidation peaks at 0.51 and 0.91 V. Insignificant anodic shifts of the oxidation potentials compared to the MWCNTs/GCE were observed that agreed with the electrochemical impedance spectroscopy data for the electrodes. The oxidation currents of both peaks were increased to 0.50 ± 0.02 and 0.42 ± 0.01 µA vs. those on the MWCNTs/GCE. This effect is caused by the higher electroactive surface area of polyFA/MWCNTs/GCE. Furthermore, the first oxidation peak was higher than the second one.

The voltammetric characteristics of hesperidin on the polyFA/MWCNTs/GCE allow its application for quantification purposes. To make a choice of the voltammetric mode and the conditions of determination, the electrooxidation of hesperidin was studied.

### 3.4. Hesperidin Electrooxidation Parameters

**Figure 7:**
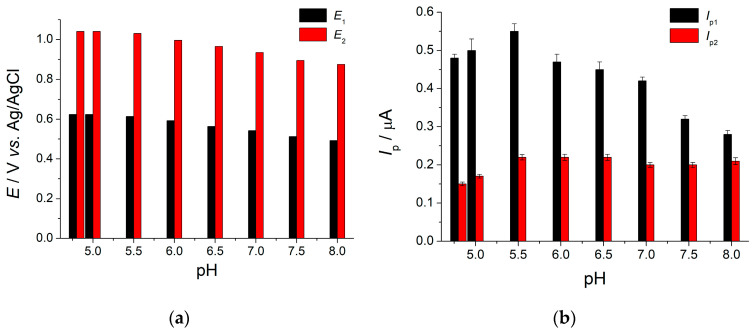
Voltammetric characteristics of 10 µmol L^−1^ hesperidin on polyFA/MWCNTs/GCE in phosphate buffer of various pH. (**a**) Oxidation potentials. (**b**) Oxidation peak currents. Data obtained by cyclic voltammetry at υ = 100 mV s^−1^.

The cyclic voltammetry of hesperidin in a phosphate buffer was studied. The effect of phosphate buffer pH in the range of 4.8−8.0 on the voltammetric response of hesperidin was evaluated. Hesperidin electrooxidation proceeded irreversibly in the whole pH range tested, since no reduction steps on the cathodic branches were observed. Both oxidation potentials became less positive with the increase in pH (Figure 7a), thereby confirming proton transfer during electrooxidation.

The oxidation currents of both peaks were increased in the pH range from 4.8 to 5.5 and then started to decrease with the pH increase. This effect can be associated with the oxidation of hesperidin by air oxygen in neutral and basic media, which is typical for flavonoids [41,42]. The highest oxidation currents were observed at pH 5.5. which was used in a further study.

**Figure 8:**
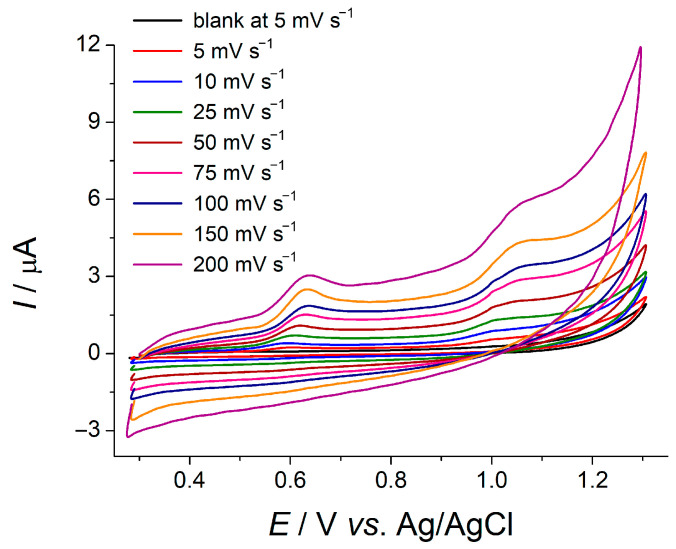
Cyclic voltammograms of 25 µmol L^−1^ hesperidin on polyFA/MWCNTs/GCE in phosphate buffer pH 5.5 at various potential scan rates.

Variation in the potential scan rate in the range of 5–200 mV s^−1^ was performed to elucidate the electrooxidation reaction of hesperidin (Figure 8). The oxidation currents of the first oxidation peak were linearly increased with the square root of the potential scan rate (Equation (2))

*I*_p1_ [µA] = (−0.07 ± 0.01) + (0.080 ± 0.001)υ^½^ [mV s^−1^]       *R*^2^ = 0.9981. (2)

The slope of the Napierian logarithmic plot (Equation (3)) was 0.58.

ln*I*_p1_ [µA] = (1.00 ± 0.03) + (0.58 ± 0.01)lnυ [V s^−1^]       *R*^2^ = 0.9979. (3)

These data made it possible to conclude that the electrooxidation of hesperidin on the polyFA-modified electrodes was a diffusion-driven process. In this case, the electrooxidation parameters (anodic transfer coefficient (α_a_), the number of electrons (*n*), diffusion coefficient (*D*), and the standard heterogeneous electron transfer rate constant (*k*^0^)) were calculated using a Tafel plot, the Randles–Ševčík equation [43], and the equation for *k*^0^ for the irreversible diffusion-controlled process [44]. The results are summarized in Table 4.

**Scheme 2:**
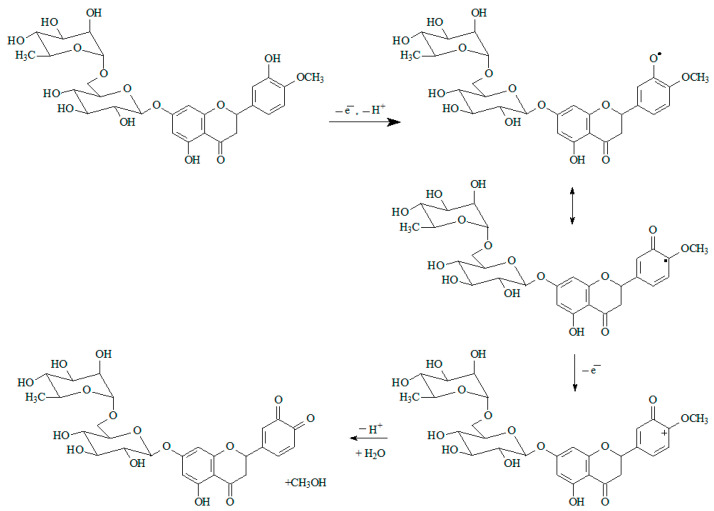
Electrooxidation of hesperidin on polyFA/MWCNTs/GCE.

The oxidation potential on the first step was anodically shifted with an increase in the scan rate, which also indicated the irreversibility of the electrode reaction of hesperidin. Thus, the irreversible two-electron electrooxidation of the hesperidin occurred on the polyFA/MWCNTs/GCE with proton participation. According to Scheme 2, electrooxidation involves ring B in the structure of hesperidin. A similar process has been reported on the MWCNTs-based electrode [45], the GCE modified with reduced graphene oxide and gold nanoparticles [46], and the GCE modified with functionalized single-walled carbon nanotubes and polyaluminon [47]. The second step on the voltammograms of the hesperidin probably corresponds to the oxidation of the hydroxyl group in ring A [47,48].

### 3.5. Sensing of Hesperidin Using PolyFA-Modified Electrode

The PolyFA/MWCNTs/GCE was used for the sensing of hesperidin under conditions of differential pulse voltammetry.

#### 3.5.1. Effect of Pulse Parameters on Hesperidin Response

**Figure 9:**
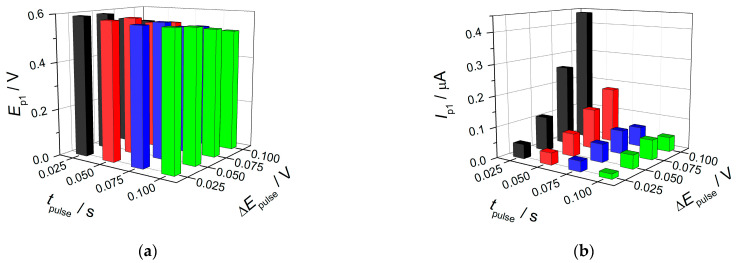
Effect of pulse parameters on the voltammetric characteristics of 1.0 µmol L^−1^ hesperidin on polyFA/MWCNTs/GCE in phosphate buffer pH 5.5. (**a**) The changes of first peak potential. (**b**) The changes of first oxidation peak currents.

Pulse parameters significantly affected the oxidation potential and currents of the hesperidin. Changes in the voltammetric characteristics were studied in 1.0 µmol L^−1^ of hesperidin. The first step oxidation potential was slowly decreased with the increase in pulse amplitude and time (Figure 9a). Oxidation peak currents grew with the increase in pulse amplitude and achieved a maximum at 0.100 V (Figure 9b). The increase in pulse time led to the decrease in hesperidin oxidation currents. Thus, a pulse amplitude of 0.100 V and a pulse time of 0.025 s provided the best response of the hesperidin and were used for its quantification.

#### 3.5.2. Differential Pulse Voltammetric Quantification of Hesperidin

**Figure 10:**
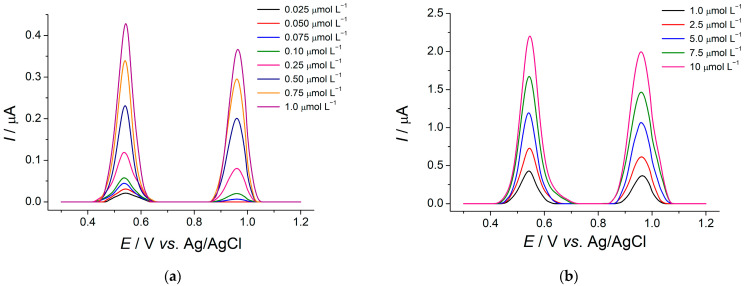
Differential pulse voltammograms with baseline correction for hesperidin on polyFA/MWCNTs/GCE in phosphate buffer pH 5.5: (**a**) 0.025–1.0 µmol L^−1^ of hesperidin; (**b**) 1.0–10 µmol L^−1^ of hesperidin. Δ*E*_pulse_ = 0.100 V, *t*_pulse_ = 0.025 s, υ = 20 mV s^−1^.

Hesperidin gave two oxidation peaks at 0.54 and 0.96 V on the differential pulse voltammograms (Figure 10). The first peak currents proportionally increased with the growth of the analyte concentration in the electrochemical cell in the ranges of 0.025–1.0 and 1.0–10 µmol L^−1^ (Figure 10a,b, respectively). The corresponding calibration graphs are presented by Equations (4) and (5), respectively

*I*_p1_ [µA] = (0.012 ± 0.001) + (42.8 ± 0.3) × 10^4^*c*_hesperidin_ [mol L^−1^]       *R*^2^ = 0.9997, (4)

*I*_p1_ [µA] = (0.250 ± 0.007) + (19.5 ± 0.1) × 10^4^*c*_hesperidin_ [mol L^−1^]       *R*^2^ = 0.9999. (5)

The high sensitivity of the hesperidin response was confirmed by the slopes of calibration graphs. The detection and quantification limits were calculated as 3SD_a_/*b* and 10SD_a_/*b*, where SD_a_ is the standard deviation of the calibration graph intercept, and *b* is the calibration graph slope. The detection and quantification limits of 7.0 and 23.4 nmol L^−1^ of hesperidin, respectively, were obtained. The analytical characteristics of the direct determination of the hesperidin on polyFA/MWCNTs/GCE are the best ones among reported to date for various electrodes, including chemically modified ones (Table 5). Furthermore, the absence of the preconcentration step in the developed method significantly reduced the measurement time and excluded the possible co-adsorption of other constituents in the case of real samples analysis. Another advantage of polyFA/MWCNTs/GCE is the ease and rapidity of fabrication.

The developed method accuracy was checked using the added–found method (Table 6). Five concentrations of hesperidin that were covered by both linear ranges were used, and recovery values of 99–100% were obtained that confirmed the high accuracy of the method. The relative standard deviation was 0.78–2.8%, and this indicates the absence of random errors in the hesperidin determination.

#### 3.5.3. Selectivity of Hesperidin Determination

The selectivity of the electrode response to hesperidin is one of the key points to be considered, since real samples usually contain a wide range of constituents, including natural phenolics of various classes. An amount of 1.0 µmol L^−1^ of hesperidin was used in the selectivity test. Typical potential interferences and structurally related phenolics were studied. The tolerance limits of the interfering species are presented in Table 7.

Inorganic cations (K^+^, Mg^2+^, Ca^2+^, and Al^3+^) and anions (NO_3_^−^, Cl^−^, and SO_4_^2−^) were electrochemically inactive in the potential range studied and did not affect the hesperidin response, even at 1000-fold excesses. Saccharides also did not oxidize under the conditions of the experiment and did not show an interference effect. Ascorbic acid is the most typical interference in citrus fruits. It did not show an oxidation signal in the potential range of 0.3–1.2 on the polyFA/MWCNTs/GCE. Ascorbic acid in addition to hesperidin did not interfere with the oxidation peaks of the hesperidin (Figure S2a).

The potential interference effects of other natural phenolics of various classes was studied. Naringin, being the second major flavanone of several citrus fruits, was oxidized at 0.75 V on the polyFA/MWCNTs/GCE (Figure S2b). A peak potential difference of 210 mV for hesperidin and naringin allowed for the determination of hesperidin in the presence of naringin, as Figure S2b shows. Caffeic and chlorogenic acids were oxidized at 0.18 V and did not affect the oxidation peak of hesperidin, (Figure S2c,d respectively). Gallic acid gave two oxidation peaks at 0.25 and 0.61 V. The second oxidation peak partially overlapped with the oxidation peak of hesperidin. However, the oxidation peak of gallic acid fully disappeared at a concentration of 10 µmol L^−1^ and less. Therefore, hesperidin determination became possible in this case (Figure S2e). Flavonols (quercetin and rutin) are also electroactive on polyFA/MWCNTs/GCE. Rutin was oxidized at 0.34 and 0.96 V. The peak potential separation with hesperidin was 200 mV and was sufficient for the registration of well-resolved peaks of both compounds (Figure S2f). Quercetin showed a quite similar effect. Its oxidation potentials were 0.25 and 0.93 V. No overlap with the hesperidin oxidation peak was observed at a quercetin concentration of 5 µmol L^−1^.

Thus, polyFA/MWCNTs/GCE shows a highly selective response to hesperidin and can be applied in the analysis of citrus fruits and products.

#### 3.5.4. Operation Characteristics of polyFA/MWCNTs/GCE

The electrode surface was mechanically renewed after each measurement (see Section 2.3.1) due to a significant decrease in the hesperidin oxidation currents after the second scan. Such behavior indicates electrode surface fouling and partial blockage of the electroactive centers. Thus, the developed electrode suggests single use only. A repeatability test for the electrode characterization was not applicable in this case.

The reproducibility of the method was considered using the relative standard deviations obtained for the accuracy test (Table 6). The values did not exceed 2.8% and showed a perfect reproducibility of the results.

The robustness of the electrode response to hesperidin was verified. The use of another GCE of the same producer, reference and auxiliary electrodes, a newly prepared suspension of MWCNTs, components of phosphate buffer, and NaOH used as a supporting electrolyte for the FA electropolymerization led to insignificant changes in the response of 0.10 µmol L^−1^ of hesperidin. The relative standard deviation of the oxidation currents was in the range of 1.0–1.5%, thereby confirming a high robustness of the developed electrode.

### 3.6. Practical Application of the Sensor in Orange Juices Analysis

Oranges are the main source of hesperidin in the human diet. Therefore, a developed sensor was applied in the analysis of fresh and commercial orange juices.

The pretreated juice samples (Section 2.3.2) showed a well-defined oxidation peak of hesperidin at 0.54 V on the differential pulse voltammograms on the polyFA/MWCNTs/GCE (Figure S3). The standard addition method confirmed that this oxidation peak belonged to hesperidin (Figure S3) for both fresh and commercial juices. The second oxidation peak at 0.74 V corresponded to naringin oxidation and agreed well with the voltammetric profile reported for orange juices [47]. A hesperidin recovery of 99.7–100.4% (Table S1) indicated the absence of matrix effects in orange juices analysis and the practical applicability of the polyFA/MWCNTs/GCE.

The quantification of hesperidin in orange juices was performed using a calibration curve and presented in Table 8. Voltammetric data were compared to those obtained with high-performance liquid chromatography with UV-detection. Good agreement of the results confirms the accuracy of the electrochemical approach. The absence of systematic errors in the quantification of the hesperidin was proven by the *t*-test values. *F*-test results indicated the insignificant difference in the results obtained by two methods, i.e., a similar precision of voltammetry and chromatography.

## 4. Conclusions

A highly sensitive and selective voltammetric sensor was developed for hesperidin quantification. The layer-by-layer combinations of MWCNTs and polyFA were shown to be an effective electrochemical platform for hesperidin detection. The electropolymerization of FA in a basic medium was performed for the first time and exhibited other roots of the electrode reaction compared to the neutral and acidic mediums. In particular, a phenolic fragment was oxidized via phenoxyl radical formation and further polymerization.

PolyFA/MWCNTs/GCE is characterized by a higher electroactive surface area and sufficient electron transfer rate to be considered as a perspective in organic electroanalysis. The analytical characteristics of thehesperidin are the best reported to date for electrochemical sensors. The selectivity of the hesperidin response in the presence of the most abundant natural phenolics allows for the application of the electrode in routine practice.

## Figures and Tables

**Table 1:** Figure of merits of electrochemical sensors based on a combination of poly(hydroxycinnamic acids) and carbon nanomaterials.

Monomer	Transducer	Method	Analyte	Linear Dynamic Range (µmol L^−1^)	Limit of Detection (µmol L^−1^)	Ref.
Caffeic acid	Carbon black/SPE ^1^	A ^2^	NADH	—	3.7	[24]
	Electrochemically reduced graphene oxide/GCE	A	Free chlorine	20–20,000	0.03	[25]
	Carbon nanotubes/GCE	CV ^3^	Glutathione	50–50,000	0.5	[26]
*p*-Coumaric acid	Multi-walled carbon nanotubes/GCE	DPV ^4^	L-Cysteine	7.5–50; 50–1000	1.1	[27]
Ferulic acid	Multi-walled carbon nanotubes/GCE	A	Dopamine	5.00–120	2.21	[28]
			Epinephrine	73.0–1406	22.28	
			NADH	59.1–1560	17.73	

^1^ Screen-printed electrode. ^2^ Amperometry. ^3^ Cyclic voltammetry. ^4^ Differential pulse voltammetry.

**Table 2:** Gradient elution program for hesperidin determination.

Time (min)	Mobile Phase Composition (% *v*/*v*)	
	Water	Acetonitrile
0	85	15
15	75	25
25	60	40
30	50	50
35	0	100
40	0	100

**Table 3:** Electrochemical impedance parameters fitted using Randles’ equivalent circuits (*n* = 5; *p* = 0.95).

Electrode	*R*_s_ (Ω)	*R*_ct_ (kΩ)	*Q* (µΩ^−1^)	*n*	*W* (µΩ^−1^)	χ^2^
Bare GCE	245 ± 5	72 ± 3	3.7 ± 0.2	0.789	—	0.029
MWCNTs/GCE	199 ± 3	12.1 ± 0.9	3.6 ± 0.1	0.775	230 ± 5	0.015
polyFA/MWCNTs/GCE	92 ± 1	21.4 ± 0.9	6.1 ± 0.3	0.751	183 ± 4	0.018

**Table 4:** Electrooxidation parameters of hesperidin on the glassy carbon electrode modified with multi-walled carbon nanotubes and poly(ferulic acid) (polyFA/MWCNTs/GCE).

Parameter	Equation	Value
α_a_	Tafel slope = α_a_*F*/2.303*RT*	0.39
*n*	∆*E*_1/2_ = 47.7/α_a_*n*	2.0
*D* (cm^2^ s^−1^)	Ip1=π12χ(bt)nFAcD12(αanαFRT)12υ12	(4.1 ± 0.3) × 10^−5^
*k*^0^ (cm s^−1^)	k0=2.415e−0.02FRTD12(Ep−Ep/2)−12υ12	2.4 × 10^−3^

**Table 5:** Analytical characteristics of the electrochemical determination of hesperidin.

Electrode	Method	Detection Limit (µmol L^−1^)	Linear Range (µmol L^−1^)	Ref.
PGE ^1^	AdSDPV ^2^	0.019	0.050–1.00	[49]
Electroactivated PGE	DPV ^3^	0.2	0.5–10	[50]
Boron-doped diamond electrode	AdSSWV ^4^	1.2	4.09–115	[51]
MWCNTs/GCE	LSV ^5^	—	0–20	[40]
MWCNTs/SPE ^6^	AdSSWV	0.0073	0–30	[45]
3D nanoporous Pt/SPE	LSV	6.61	10–400	[52]
SnO_2_ nanoparticles in cetylpyridium bromide/GCE	AdDPV ^7^	0.077	0.10–10; 10–75	[53]
Mesoporous SiO_2_ modified CPE ^8^	AdDPV	0.25	0.5–25	[54]
Brilliant-green/Nano-graphene-platelet/CPE	DPV	0.050	0.10–7.0; 7.0–100	[55]
Au nanoparticles/electro-reduced graphene oxide/GCE	A ^9^	0.0082	0.050–8.0	[46]
Polyaluminon/f-SWCNTs ^10^/GCE	DPV	0.029	0.10–2.5; 2.5–25	[47]
Poly(*o*-aminothiophenol)/Au nanoparticles/Ultrafine activated carbon/GCE	DPV	0.045	0.085–30	[56]
PolyFA/MWCNTs/GCE	DPV	0.007	0.025–1.0; 1.0–10	This work

^1^ Pencil graphite electrode. ^2^ Adsorptive stripping differential pulse voltammetry. ^3^ Differential pulse voltammetry. ^4^ Adsorptive stripping square-wave voltammetry. ^5^ Linear sweep voltammetry. ^6^ Screen-printed electrode. ^7^ Adsorptive differential pulse voltammetry. ^8^ Carbon paste electrode. ^9^ Amperometry. ^10^ Polyaminobenzene sulfonic acid functionalized single-walled carbon nanotubes.

**Table 6:** Hesperidin quantification in model systems using polyFA/MWCNTs/GCE in phosphate buffer pH 5.5 (*n* = 5; *p* = 0.95).

Added (µmol L^−1^)	Found (µmol L^−1^)	RSD (%)	*R* (%)
0.025	0.0250 ± 0.0009	2.8	100 ± 3.5
0.25	0.248 ± 0.005	1.5	99 ± 2
1.0	1.00 ± 0.02	1.5	100 ± 2
5.0	4.98 ± 0.05	0.78	99.7 ± 0.9
10	10.0 ± 0.1	1.1	100 ± 1

**Table 7:** Tolerance limits of interference for the determination of 1.0 µmol L^−1^ hesperidin on the polyFA/MWCNTs/GCE in phosphate buffer pH 5.5 (*n* = 5; *p* = 0.95).

Interfering Specimen	Tolerance Limit (µmol L^−1^)	Hesperidin Recovery (%)
K^+^, Mg^2+^, Ca^2+^, and Al^3+^	1000	100 ± 1
NO_3_^−^, Cl^−^, and SO_4_^2−^	1000	100 ± 1
Glucose	100	99.9 ± 0.2
Sucrose	100	100 ± 1
Rhamnose	100	100 ± 1
Ascorbic acid	100	100 ± 2
Naringin	100	100.2 ± 0.3
Caffeic acid	100	99.8 ± 0.2
Chlorogenic acid	100	100 ± 2
Gallic acid	10	99.5 ± 0.5
Quercetin and rutin	5.0	100 ± 2

**Table 8:** Quantification of hesperidin in orange juices (*n* = 5; *p* = 0.95).

Juice Type	Sample	Hesperidin Contents (mg L^−1^)				*t*-Test ^1^	*F*-Test ^2^
		Voltammetry	RSD (%)	Chromatography	RSD (%)		
Fresh	1	172 ± 4	1.6	170 ± 6	1.4	0.970	1.34
Commercial	2	112 ± 2	1.3	113 ± 4	1.6	0.912	1.56
	3	211 ± 4	1.2	212 ± 6	1.1	0.358	1.29
	4	251 ± 4	1.1	252 ± 8	1.3	0.218	1.50

^1^ *t*_crit_ = 2.45 at α = 0.05 and *f* = 6. ^2^ *F*_crit_ = 19.25 at α = 0.05 and *f*_1_ = 4 and *f*_2_ = 2.

## Data Availability

Data are contained within the article and Supplementary Materials.

## Supplementary Materials

The following supporting information can be downloaded at: https://www.mdpi.com/article/10.3390/bios13050500/s1, Figure S1: Hesperidin structure; Figure S2: Differential pulse voltammograms with baseline correction for the mixtures of hesperidin with potential interferences on polyFA/MWCNTs/GCE in phosphate buffer pH 5.5: (**a**) 1.0 µmol L^−1^ of hesperidin and 100 µmol L^−1^ of ascorbic acid; (**b**) 1.0 µmol L^−1^ of hesperidin and 100 µmol L^−1^ of naringin; (**c**) 1.0 µmol L^−1^ of hesperidin and 100 µmol L^−1^ of caffeic acid; (**d**) 1.0 µmol L^−1^ of hesperidin and 100 µmol L^−1^ of chlorogenic acid; (**e**) 1.0 µmol L^−1^ of hesperidin and 10 µmol L^−1^ of gallic acid; (**f**) 1.0 µmol L^−1^ of hesperidin and 5 µmol L^−1^ of rutin. Δ*E*_pulse_ = 0.100 V, *t*_pulse_ = 0.025 s, υ = 20 mV s^−1^; Figure S3: Typical differential pulse voltammograms with baseline correction for orange juice on polyFA/MWCNTs/GCE in phosphate buffer pH 5.5: (**a**) 10 µL of commercial juice with various additions of hesperidin; (**b**) 10 µL of fresh orange juice with various additions of hesperidin. Δ*E*_pulse_ = 0.100 V, *t*_pulse_ = 0.025 s, υ = 20 mV s^−1^; Table S1: Recovery of hesperidin in orange juice (*n* = 5; *p* = 0.95).
