# Supplementary material for: Layer-by-Layer Combination of MWCNTs and Poly(ferulic acid) as Electrochemical Platform for Hesperidin Quantification
Source: Biosensors (Basel). 2023 Apr 25;13(5):500. doi: 10.3390/bios13050500 (PMC10216161; doi:10.3390/bios13050500)
Supplement: Supplementary file 1 [file biosensors-13-00500-s001.zip › biosensors-2279030-supplementary.pdf]

Supplementary Material

Layer-by-Layer Combination of MWCNTs and Poly(Ferulic Acid) as Electrochemical Platform for Hesperidin Quantification

Elvira Yakupova [1,2], Aisylu Mukharlyamova [2], Igor Fitsev [2] and Guzel Ziyatdinova [1,*]

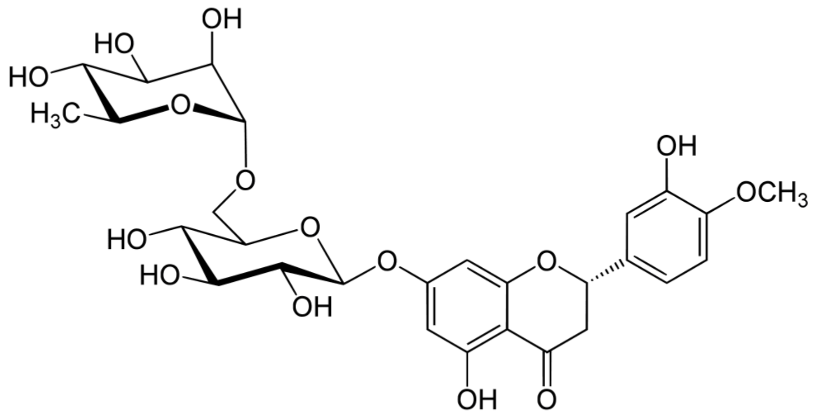

Figure S1. Hesperidin structure.

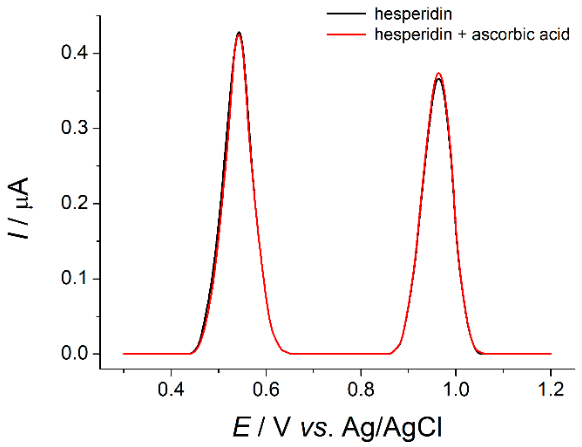

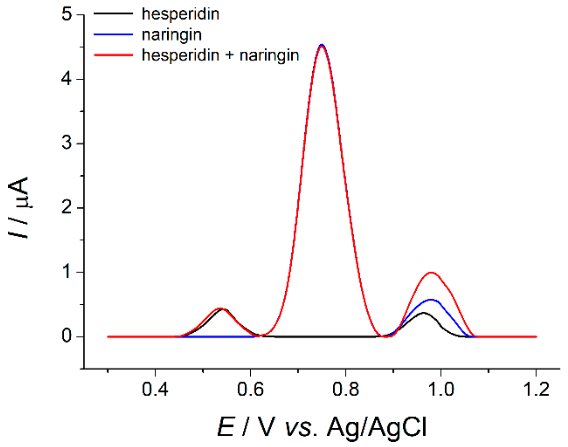

(a) (b)

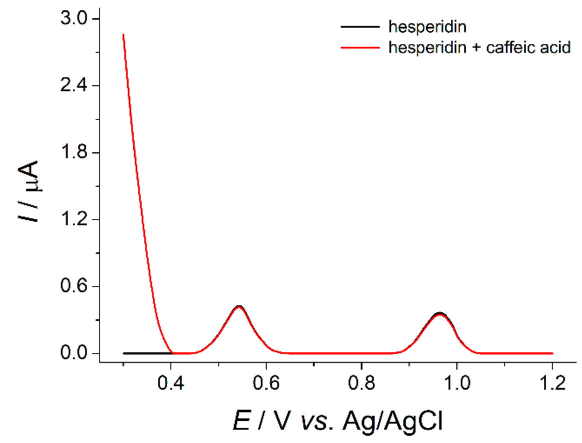

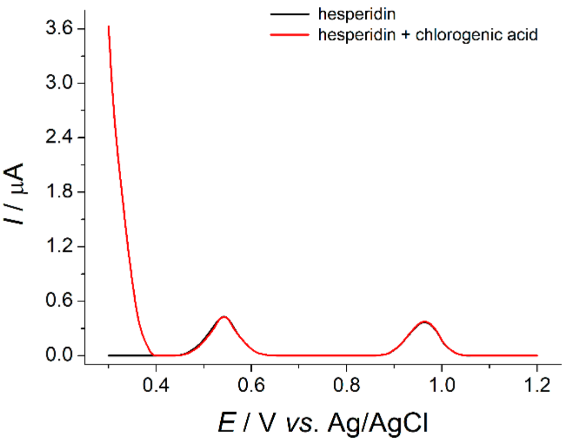

(c)

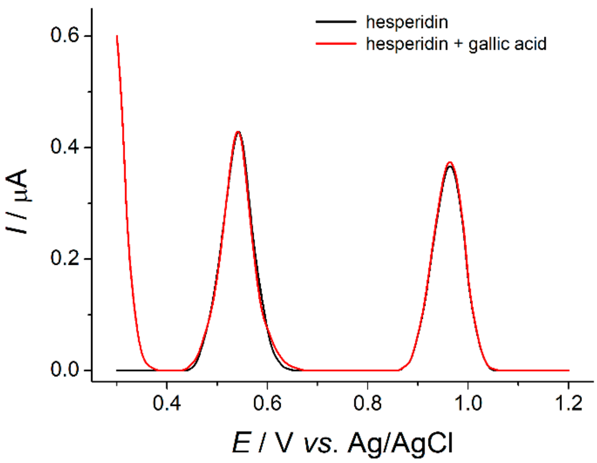

(d)

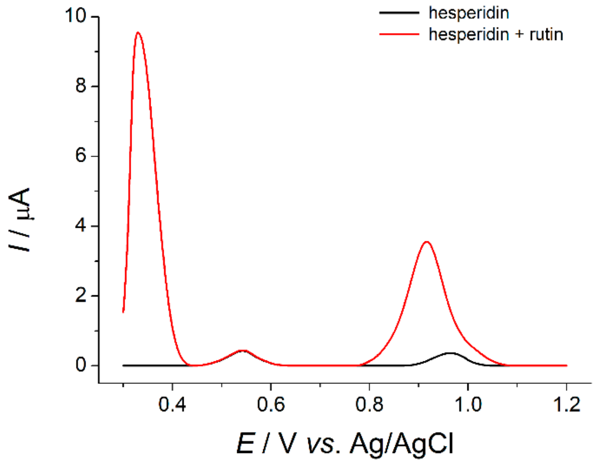

(e)

(f)

Figure S2. Differential pulse voltammograms with baseline correction for the mixtures of hesperidin with potential interferences on polyFA/MWCNTs/GCE in phosphate buffer pH 5.5: (**a**) 1.0 µmol L^{-1} of hesperidin and 100 µmol L^{-1} of ascorbic acid; (**b**) 1.0 µmol L^{-1} of hesperidin and 100 µmol L^{-1} of naringin; (**c**) 1.0 µmol L^{-1} of hesperidin and 100 µmol L^{-1} of caffeic acid; (**d**) 1.0 µmol L^{-1} of hesperidin and 100 µmol L^{-1} of chlorogenic acid; (**e**) 1.0 µmol L^{-1} of hesperidin and 10 µmol L^{-1} of gallic acid; (**f**) 1.0 µmol L^{-1} of hesperidin and 5 µmol L^{-1} of rutin. ΔE_{pulse} = 0.100 V, t_{pulse} = 0.025 s, υ = 20 mV s^{-1}.

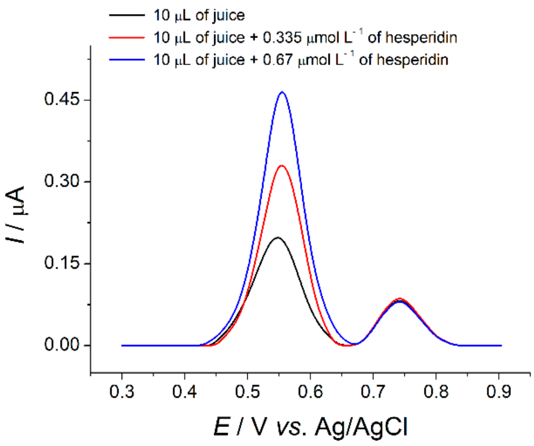

(a)

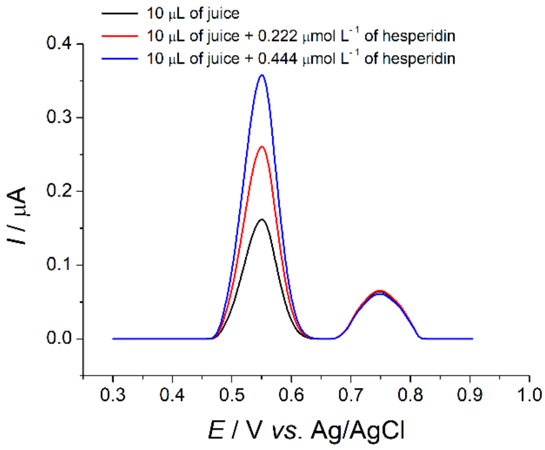

(b)

Figure S3. Typical differential pulse voltammograms with baseline correction for orange juices on polyFA/MWCNTs/GCE in phosphate buffer pH 5.5: (**a**) 10 µL of commercial juice with various additions of hesperidin; (**b**) 10 µL of orange fresh with various additions of hesperidin. ΔE_{pulse} = 0.100 V, t_{pulse} = 0.025 s, υ = 20 mV s^{-1}.

Table S1. Recovery of hesperidin in orange juices (n = 5; p = 0.95).

Sample	Spiked (µmol L^{-1})	Found (µmol L^{-1})	RSD (%)	Recovery (%)
	0	0.352 ± 0.008	1.5	
Orange fresh	0.222	0.572 ± 0.009	1.2	99.7 ± 1.5
	0.444	0.794 ± 0.009	0.92	99.7 ± 1.1
	0	0.432 ± 0.008	1.2	
entry 3	0.335	0.77 ± 0.01	1.2	100.4±1.3
	0.670	1.10 ± 0.06	2.3	100.2 ± 2.0
